# Author Correction: Discovery of indole-modified aptamers for highly specific recognition of protein glycoforms

**DOI:** 10.1038/s41467-022-31223-5

**Published:** 2022-06-15

**Authors:** Alex M. Yoshikawa, Alexandra Rangel, Trevor Feagin, Elizabeth M. Chun, Leighton Wan, Anping Li, Leonhard Moeckl, Diana Wu, Michael Eisenstein, Sharon Pitteri, H. Tom Soh

**Affiliations:** 1 Department of Chemical Engineering, Stanford University, Stanford, CA 94305 USA; 2 Department of Radiology, Stanford University, Stanford, CA 94305 USA; 3 Department of Bioengineering, Stanford University, Stanford, CA 94305 USA; 4 Department of Chemistry, Stanford University, Stanford, CA 94305 USA; 5 Department of Electrical Engineering, Stanford University, Stanford, CA 94305 USA; 6 Chan Zuckerberg Biohub, San Francisco, CA 94158 USA

**Keywords:** High-throughput screening, Glycobiology

Correction to: *Nature Communications* 10.1038/s41467-021-26933-1, published online 07 December 2021.

In this article the author name Leonhard Moeckl was incorrectly written as Leonhard Moekl. The original article has been corrected.

