# Supplementary material for: Discovery of indole-modified aptamers for highly specific recognition of protein glycoforms
Source: Nat Commun. 2021 Dec 7;12:7106. doi: 10.1038/s41467-021-26933-1 (PMC8651674; doi:10.1038/s41467-021-26933-1)
Supplement: Supplementary file 2 — Supplementary Information [file 41467_2021_26933_MOESM2_ESM.pdf]

Supplemental information

Image processing code:

```
newimgUB = MinDetect[ImageAdjust[unblockedImage, 1], .2] // MorphologicalTransform[#,"Fill"] &;
circlesUB = ComponentMeasurements[
newimgUB, {"Image","Area","Count","Mean","StandardDeviation"},#Count>5 &];
Histogram@circlesUB[[All,2,3]];


newimgBL = MinDetect[ImageAdjust[blockedImage, 1], .2] // MorphologicalTransform[#,"Fill"] &;
circlesBL = ComponentMeasurements[
newimgBL, {"Image","Area","Count","Mean","StandardDeviation"},#Count>5 &];
Histogram@circlesBL[[All,2,3]];

fivexHisto=Histogram[{circlesUB[[All,2,3]],circlesBL[[All,2,3]]},
    {3},
    PlotRange→{{0,100},{0,600}},
    ImageSize→250,
    BaseStyle→Directive[FontFamily→"Arial",FontSize→12],
    Frame→{True,True,False,False},
    FrameLabel→{Style["Particle Size",12, FontFamily→"Arial"],Style["Count",12,FontFamily→"Arial"]},
    ChartBaseStyle→Automatic
]
```

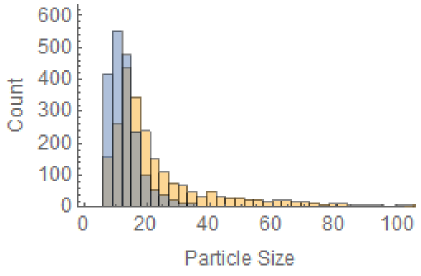

Supplementary Tables:

Name	Sequence
i-1	ZZZGZGCGCZZCAGZCAAZZGGCAZZCGZGAAZAZCZCCZZZZACGACAC
i-2	ZCGGZGGACGCCZCZZGAZCGAGZZZZGGZCAZGZGZZZACGZAZGZZZC
i-3	GCGGZZCZZACZCAGCCCZAZGCZACGACACZCZZZCZZACCCCAACCZA
i-4	ZZZGZGCGCZZCAGZCAAZZGGCAZZCGZGAAZAZCZCCZZZZZACGACA
i-5	CGGZZCZZACZCAGCCCZAZGCZACGACACZCZZZCZZACCCCAACCZAC
i-6	AZCZCGZGZGZZZGGZZZZAGAZZZZZGGAAAZZAZGAZZCCZGZZGGZC
i-7	ZAGCAZGZCAGZCAACCZZCAGZAZGCZCGZZGZGAZZZZZZZCGCAZAC
i-6scr	GZZAZZAZZZAZZACZGGGZCZZAGAGZZCGZZZZGAGZZGGZGCACZZZ

Supplementary Table 1: RB aptamer sequences selected from HTS data. Primer regions not shown. The indole-modified dUTP analog is represented by the letter Z.

Name	Sequence
f-1	CAAAGCCACZGAACCACCCGGCGGAAAAAGAGAZGGAGCA
f-2	AGAAAAGCGAGAAACCGGAGGGAGCGCGGCAACCGACA
f-3	CAAAAAGAAACCAGCAAZCAAAACCCGCAAGCAGCAAZAG
f-4	CGCCAAAGCZCGAZCAAZAACCGAAAGAAAAGGCACAZCA
f-5	CCGGGCCACGAACZCAAZGGAGACGCACGCCAAACACGGA
f-6	CAGAAAAAGGGAGZGGGZGGGZCGGGCAGAGGAAGGGGAG
f-7	CAGAAGAAGZGAGZGGGZGGGZCGGGCAGAGGAAGGGGAG

Supplementary Table 2: Fetuin aptamer sequences selected from HTS. Indole-modified dUTP analogs are represented by the letter Z.

Supplementary Figures:

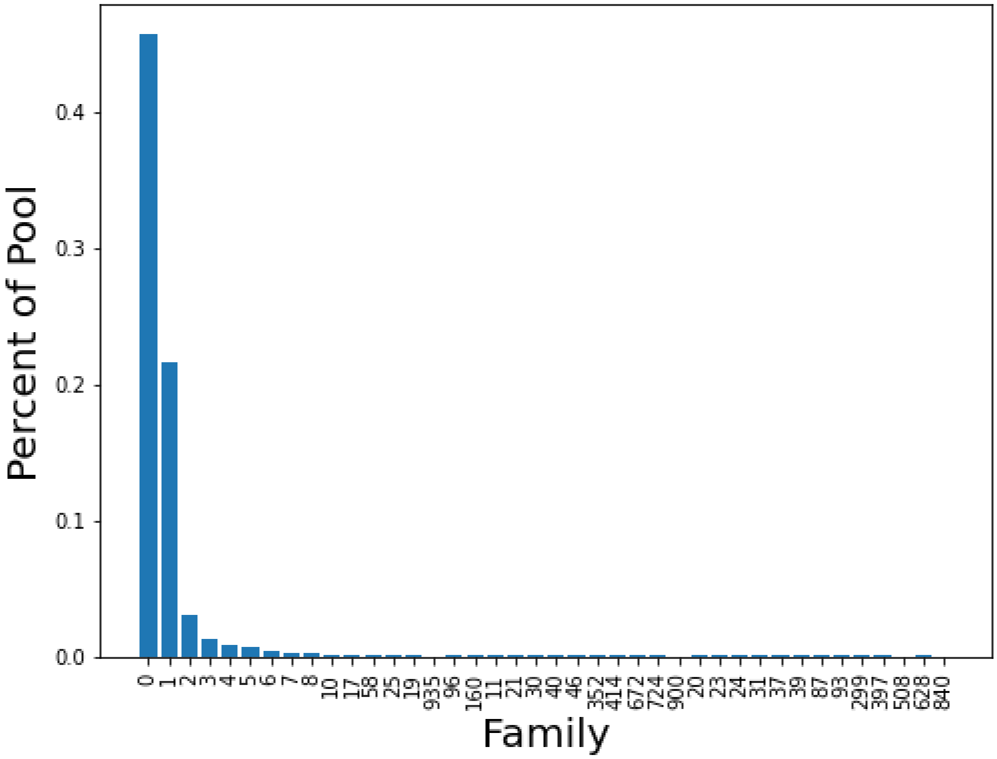

Supplementary Figure 1: Abundance of RB aptamer families. Aptamer families were identified from the HTS data after MPPD selection and were defined as sequences with a Levenshtein distance ≤ 3.

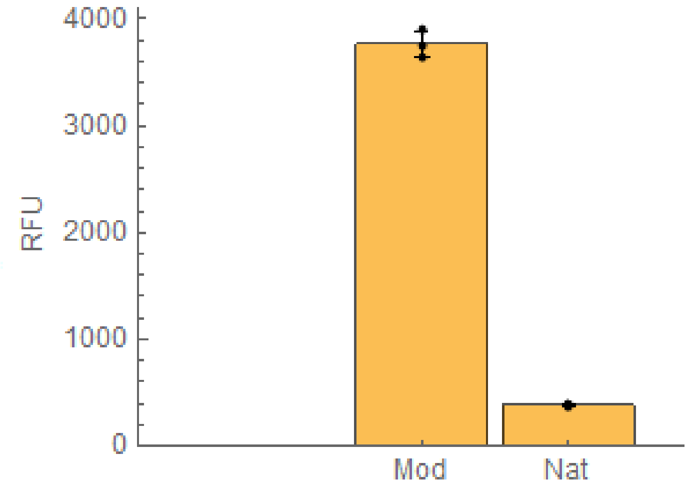

Supplementary Figure 2: Binding of base-modified and natural DNA versions of aptamer i-6 to 100 μM fluorescently-labeled RB. Experiments were conducted in triplicate and the error bars represent the standard deviation of the median RFU values. The raw data points for each experiment are overlayed onto the bar plot as black dots.

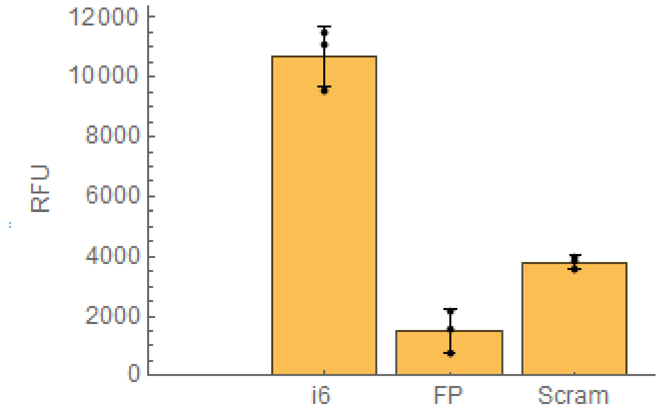

Supplementary Figure 3: Plot of fluorescence from bead-based binding assays of i-6 aptamer particles, forward primer (FP)-coated beads, and aptamer particles coated with a scrambled i-6 aptamer sequence (Scram) after incubation with 20 μM fluorescently-labeled RB. Experiments were conducted in triplicate and error bars represent the standard deviation of the median RFU values. The raw data points for each experiment are overlayed onto the bar plot as black dots.

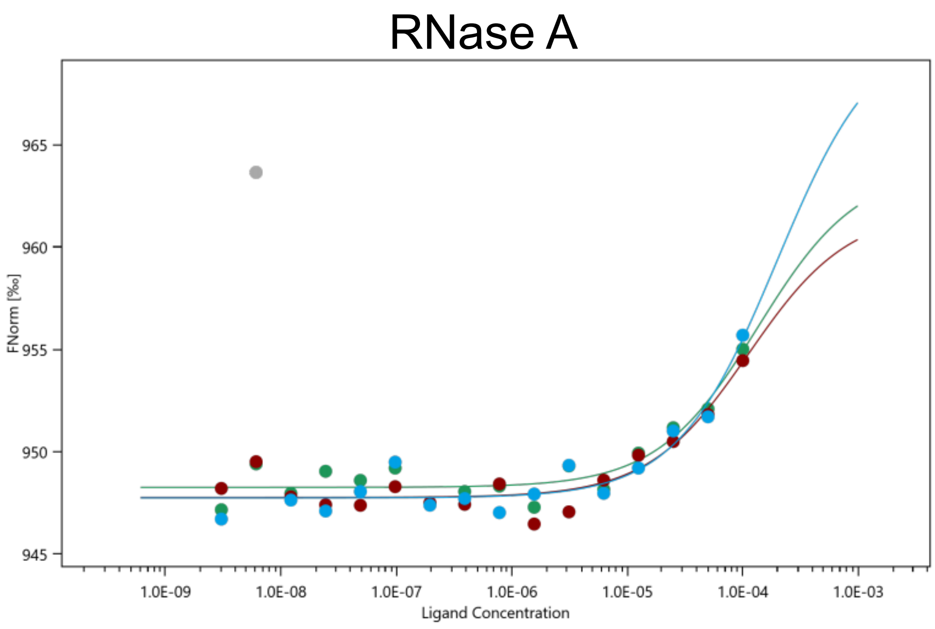

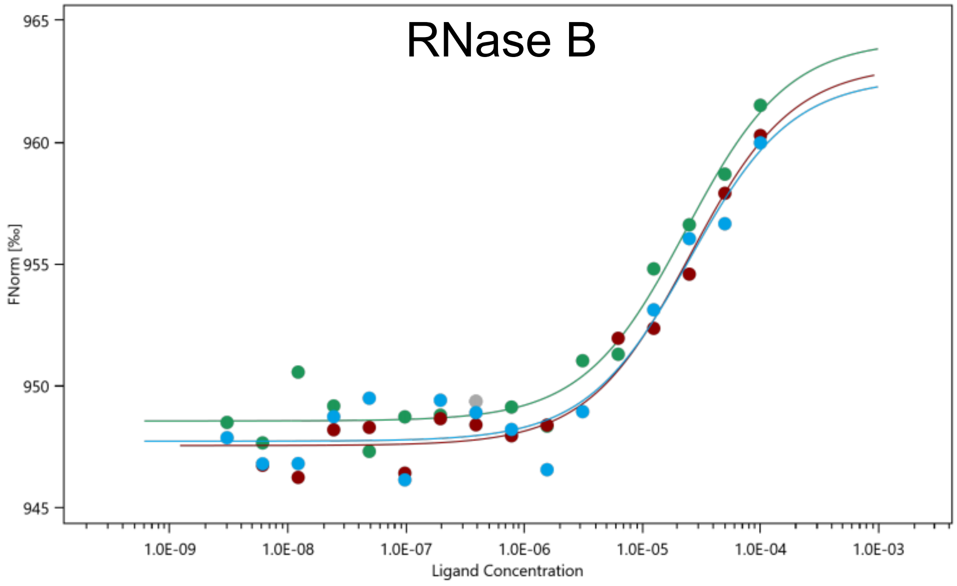

Supplementary Figure 4: MST binding affinity experiments with aptamer i-6 5'-labeled with Cy5 and unlabeled RA (top) or RB (bottom). Each experiment was conducted in triplicate, and each replicate consisted of a separate incubation using the same stock of protein and Cy5 labeled aptamer. The lines represent the fits for a binary binding model as fitted by the MO Affinity Analysis software (K_D = 148.2 µM ± 44.6 µM for RA and 24.5 µM ± 1.2 µM for RB).

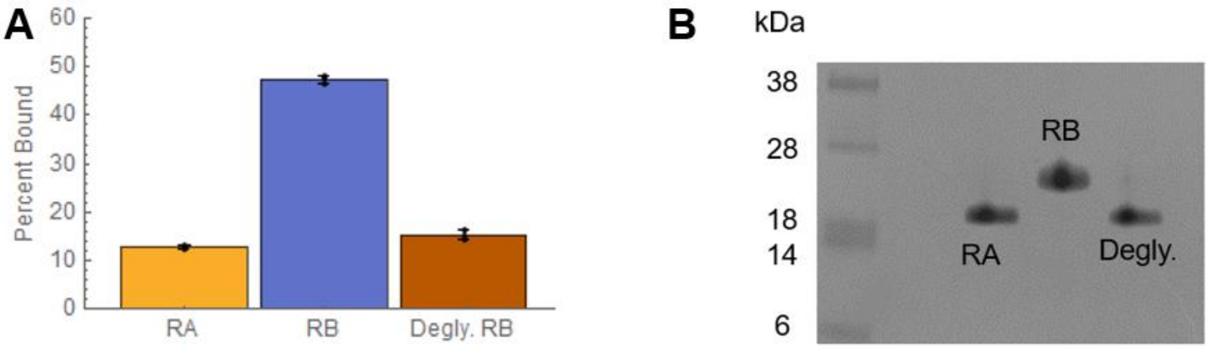

Supplementary Figure 5: A) Bead-based binding assays for i-6 aptamer beads with 20 μM fluorescently-labeled RA, RB, or deglycosylated RB (via treatment with PNGase F). Experiments were conducted in triplicate, and percent bound was determined from the median RFU values. Error bars represent a single standard deviation. The raw data points for each experiment are overlayed onto the bar plot as black dots. **B)** SDS-PAGE analysis of RA, RB, and deglycosylated RB. Uncropped gel image is available in the Source Data file.

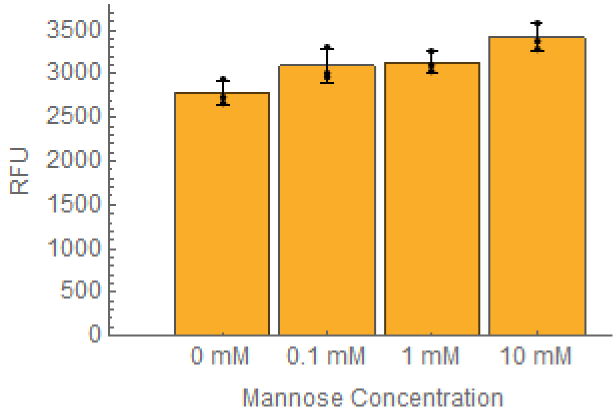

Supplementary Figure 6: Competitive binding assays for i-6 aptamer particles and 20 µM fluorescently-labeled RB with various concentrations of mannose. Experiments were conducted in triplicate, and error bars represent the standard deviation of the median RFU values. The raw data points for each experiment are overlayed onto the bar plot as black dots.

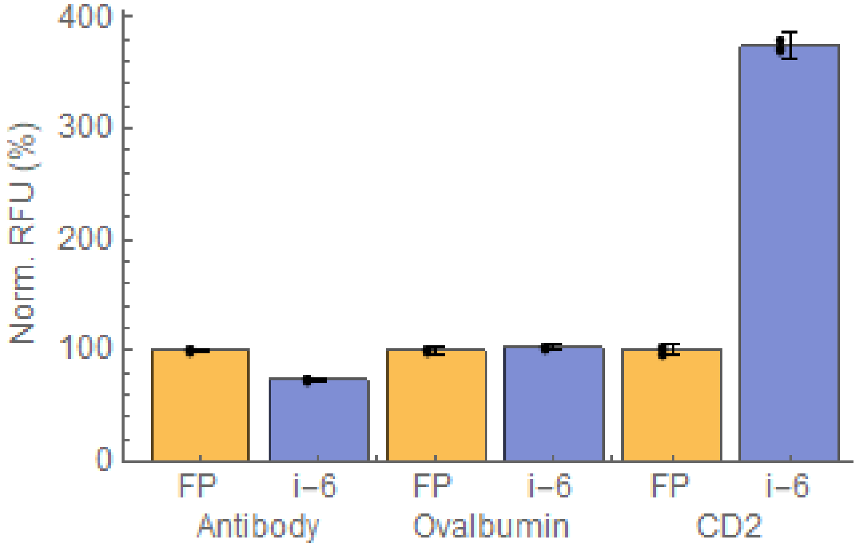

Supplementary Figure 7: Aptamer i-6 and FP bead glycoprotein binding assays. Flow-cytometry binding assays with FP beads and i-6 aptamer particles were conducted with either 10 µM FITC-labeled polyclonal goat antibody, 50 µM FITC-labeled ovalbumin, or 25 µM Dylight-655 labeled CD2. Median RFU values were taken and normalized as a percentage of the FP signal. Experiments were conducted in triplicate, and the error bars represent the standard deviation of the median RFU values. The raw data points for each experiment are overlayed onto the bar plot as black dots.

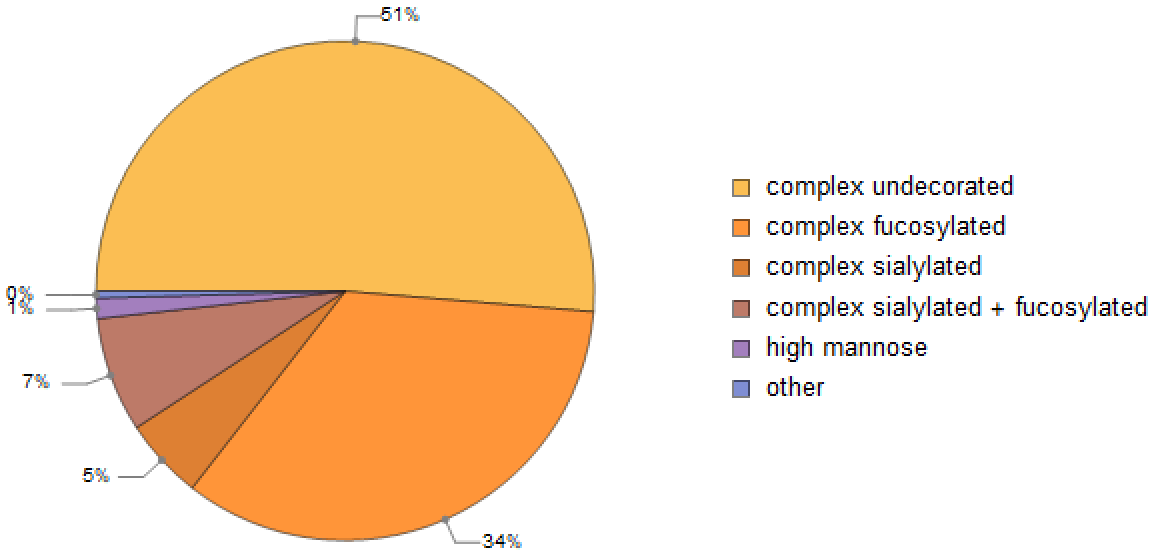

Supplementary Figure 8: Mass-spectroscopy analysis of ovalbumin N-glycan composition. The percent of each type of N-glycan is shown. Detailed results of the mass spectroscopy analysis are included as an additional file titled “Mass Spectrometry Data”.

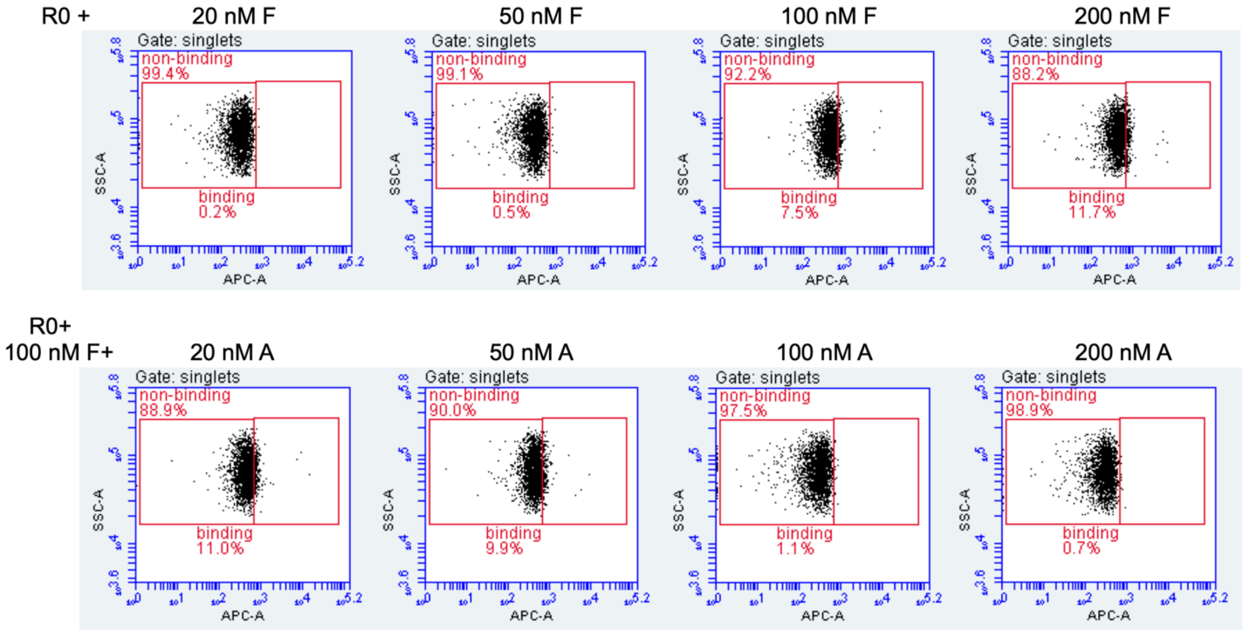

Supplementary Figure 9: Flow cytometry-based binding assay of the pre-enriched library (R0) prior to MPPD screening. An initial assay was performed to evaluate binding of the library to various concentrations of Dylight-650-labeled fetuin (F; top) to determine which concentration resulted in a sufficient shift in the APC channel. Based on these results, we performed a second assay with 100 nM Dylight-650 labeled fetuin to determine how much binding was retained in the presence of increasing concentrations of AlexaFluor 532-labeled asialofetuin (A). A final concentration of 100 nM fetuin and 200 nM asialofetuin was chosen for two-color FACS.

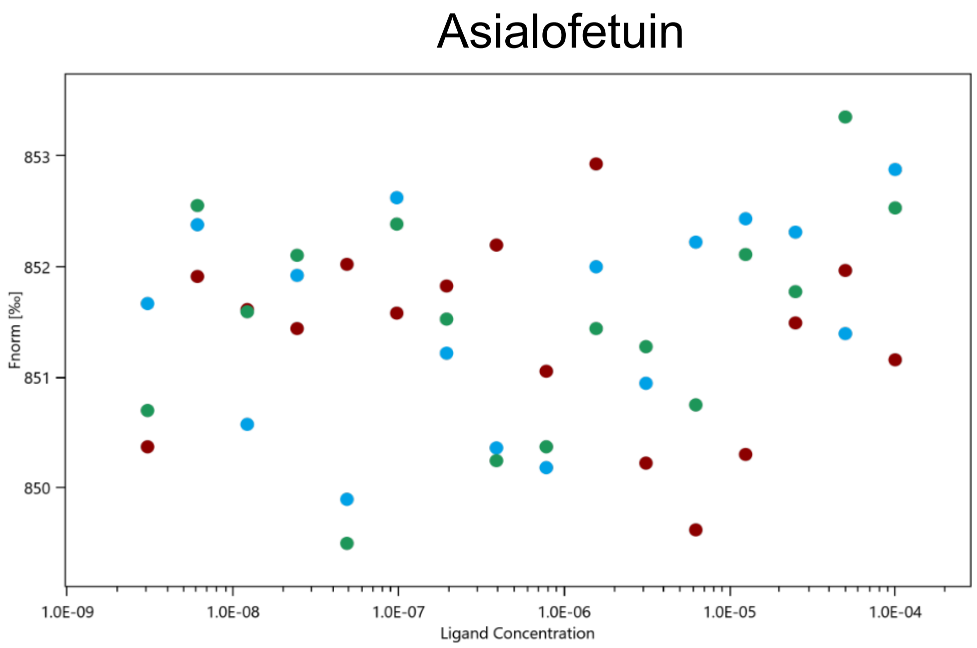

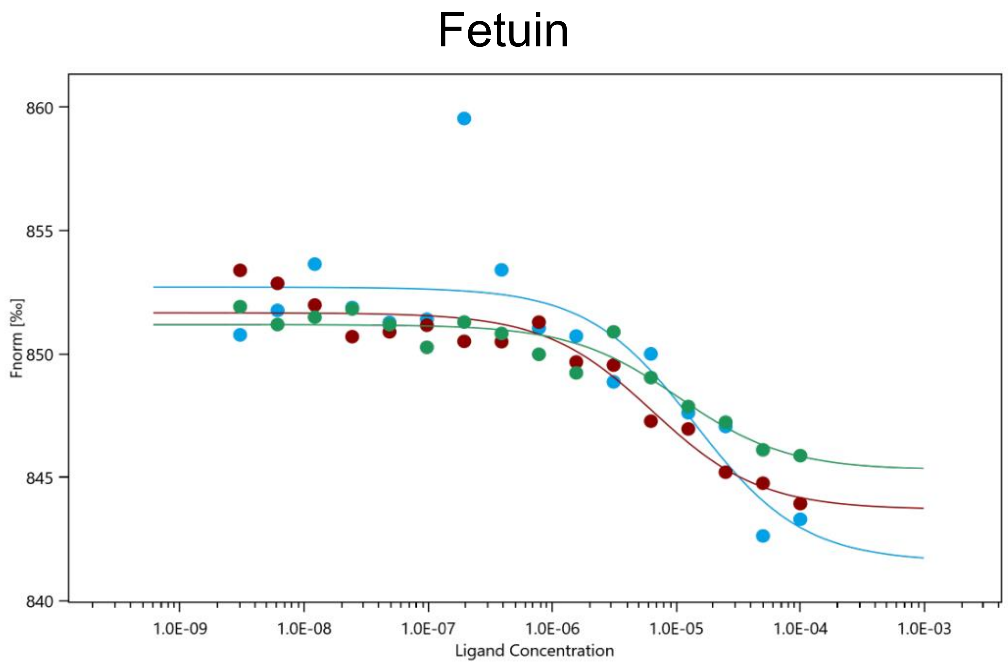

Supplementary Figure 10: MST binding affinity experiments using Cy5-labeled aptamer f-4 and unlabeled asialofetuin (top) or fetuin (bottom). Each experiment was conducted in triplicate, and each replicate consisted of a separate incubation using the same stock of protein and Cy5-labeled aptamer. The lines represent the fits for a binary binding model as fitted by the MO Affinity Analysis software (no binding detected for asialofetuin; K_D = 10.4 µM ± 3.9 µM for fetuin).

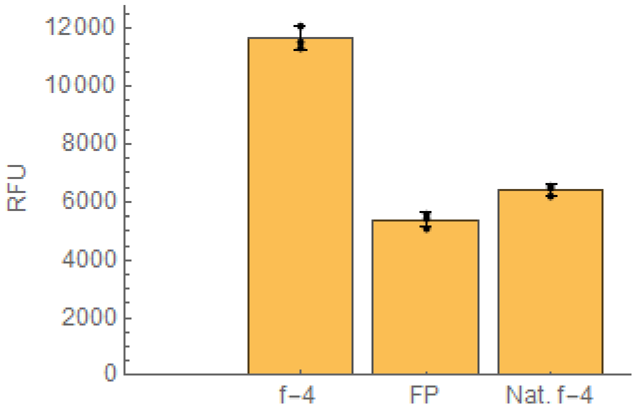

Supplementary Figure 11: Bead-based binding assays with 20 μM fluorescently-labeled fetuin and aptamer particles coated with f-4 aptamer, forward primer (FP), or a natural DNA version of f-4 with no indole modifications. Experiments were conducted in triplicate, and the error bars represent the standard deviation of the median RFU values. The raw data points for each experiment are overlayed onto the bar plot as black dots.

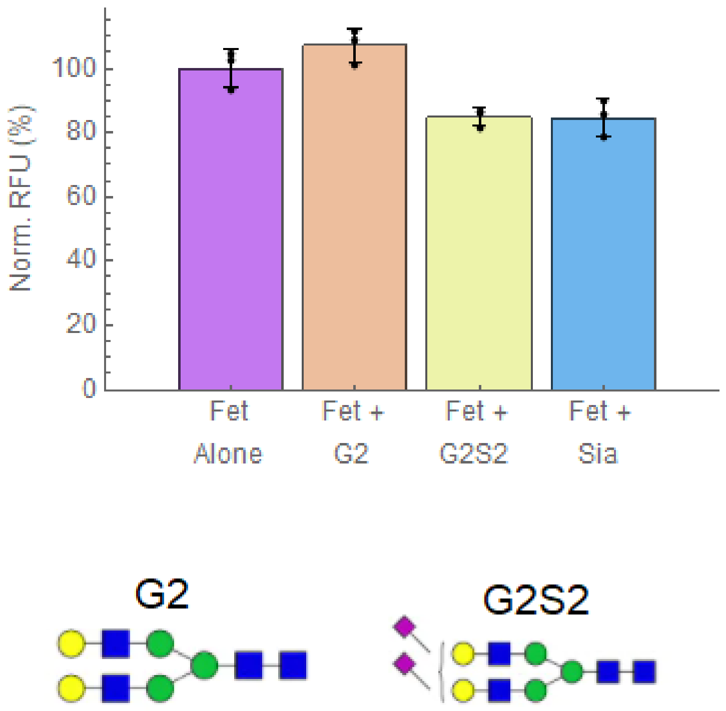

Supplementary Figure 12: Aptamer f-4-fetuin competition assays with various glycans. Flow cytometry assays conducted with f-4 aptamer particles against 20 µM fluorescently-labeled fetuin alone or in competition with 50 µM G2, 50 µM G2S2, or 100 µM sialic acid. Experiments were conducted in triplicate, and the error bars represent the standard deviation of the median RFU values. The raw data points for each experiment are overlayed onto the bar plot as black dots.

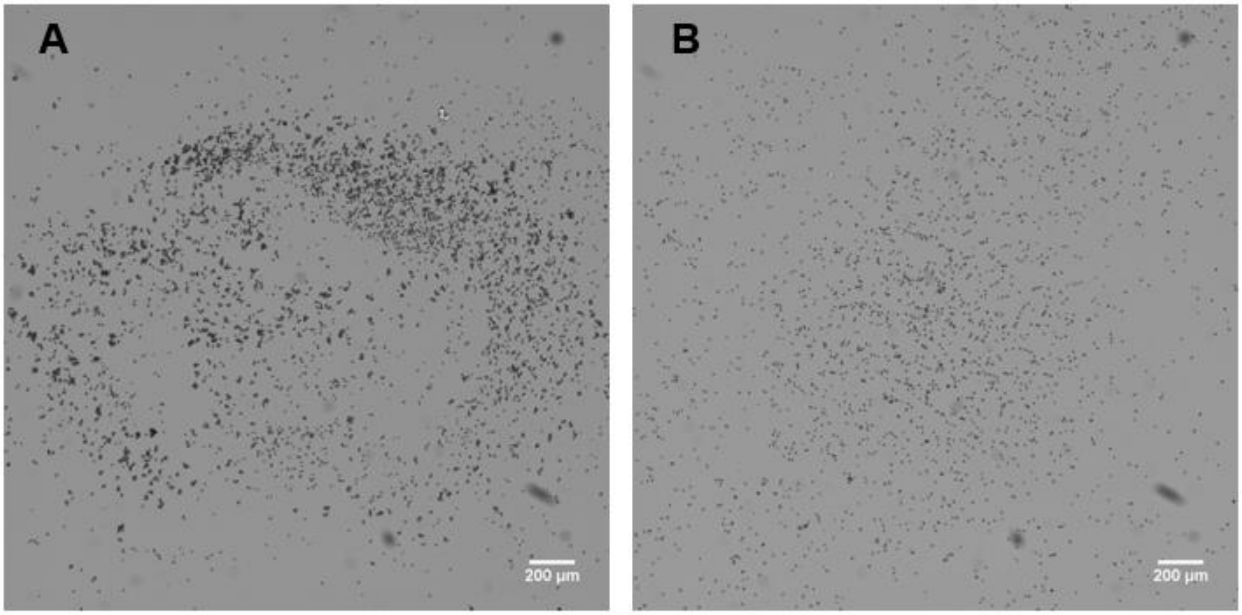

Supplementary Figure 13: Aptamer i-6 bead-based cell adhesion assays with **A)** unblocked or **B)** ConA-blocked fixed cells. Images were collected with a 5X objective and scale bars represent 200 microns. Each experimental condition was replicated once with similar results.
