# Supplementary material for: Discovery of indole-modified aptamers for highly specific recognition of protein glycoforms
Source: Nat Commun. 2021 Dec 7;12:7106. doi: 10.1038/s41467-021-26933-1 (PMC8651674; doi:10.1038/s41467-021-26933-1)
Supplement: Supplementary file 3 — Description of Additional Supplementary Files [file 41467_2021_26933_MOESM3_ESM.pdf]

Title: Supplementary Data 1

Description: Ovalbumin glycopeptides identified by LC-MS/MS analysis. Glycopeptides are classified by putative glycan type including: complex undecorated (no fucose or sialic acid), complex fucosylated, complex sialylated, complex fucosylated + sialylated, high mannose, or other (HexNAc2).
