# Supplementary material for: Discovery of indole-modified aptamers for highly specific recognition of protein glycoforms
Source: Nat Commun. 2021 Dec 7;12:7106. doi: 10.1038/s41467-021-26933-1 (PMC8651674; doi:10.1038/s41467-021-26933-1)
Supplement: Supplementary file 5 — Reporting Summary [file 41467_2021_26933_MOESM5_ESM.pdf]

nature research

Corresponding author(s): Hyongsok Tom Soh

Last updated by author(s): Oct 13, 2021

Reporting Summary

Nature Research wishes to improve the reproducibility of the work that we publish. This form provides structure for consistency and transparency in reporting. For further information on Nature Research policies, see our Editorial Policies and the Editorial Policy Checklist.

Statistics

For all statistical analyses, confirm that the following items are present in the figure legend, table legend, main text, or Methods section.

n/a	Confirmed	
☐	☒	The exact sample size (n) for each experimental group/condition, given as a discrete number and unit of measurement
☐	☒	A statement on whether measurements were taken from distinct samples or whether the same sample was measured repeatedly
☒	☐	The statistical test(s) used AND whether they are one- or two-sided<br>*Only common tests should be described solely by name; describe more complex techniques in the Methods section.*
☒	☐	A description of all covariates tested
☒	☐	A description of any assumptions or corrections, such as tests of normality and adjustment for multiple comparisons
☐	☒	A full description of the statistical parameters including central tendency (e.g. means) or other basic estimates (e.g. regression coefficient) AND variation (e.g. standard deviation) or associated estimates of uncertainty (e.g. confidence intervals)
☒	☐	For null hypothesis testing, the test statistic (e.g. F, t, r) with confidence intervals, effect sizes, degrees of freedom and P value noted<br>*Give P values as exact values whenever suitable.*
☒	☐	For Bayesian analysis, information on the choice of priors and Markov chain Monte Carlo settings
☒	☐	For hierarchical and complex designs, identification of the appropriate level for tests and full reporting of outcomes
☒	☐	Estimates of effect sizes (e.g. Cohen's d, Pearson's r), indicating how they were calculated

Our web collection on statistics for biologists contains articles on many of the points above.

Software and code

Policy information about availability of computer code

Data collection	Micro-manager (version 1.4) was used to collect microscope images. For sorting beads, BD FACSDiva software (version 8.0.2) was used.
Data analysis	Wolfram Mathematica 12 (version 12.0.0.0) was used to create the bar chart plots, binding curves, calculate statistics, and to analyze the cell-adhesion images. The Mathematica code used to analyze the cell-adhesion assays is available in the SI. FlowJo (version 10.6) was used to generate Fig. 3. ImageJ (version 1.52a) was used to manually inspect the cell-adhesion images. PyMOL (version 2.3.4) was used to generate Fig 2A. Python (version 3.9.0) was used for the aptamer family analysis using the levenshtein_distance function from the Levenshtein Python library (version 0.12.2). For the MST experiments MO.Control v2.1 and MO.Affinity Analysis v3.0.4 were used. For analysis of binding assays, BD Accuri C6 Plus Software (version 1.0.23.1) was used. The raw mass spectrometry data files were searched using Byonic software 4.0.12 (Protein Metrics).

For manuscripts utilizing custom algorithms or software that are central to the research but not yet described in published literature, software must be made available to editors and reviewers. We strongly encourage code deposition in a community repository (e.g. GitHub). See the Nature Research guidelines for submitting code & software for further information.

Data

Policy information about availability of data

All manuscripts must include a data availability statement. This statement should provide the following information, where applicable:

- Accession codes, unique identifiers, or web links for publicly available datasets
- A list of figures that have associated raw data
- A description of any restrictions on data availability

The data underlying Figures 4–6 as well as SI Figures 2, 3, 4, 5, 6, 7, 10, 11, and 12 are contained in the Source Data file. The mass spectrometry data for the

ovalbumin glycoprotein is contained in the Mass Spectrometry file. Any additional data from this study is available from the authors upon reasonable request.

Field-specific reporting

Please select the one below that is the best fit for your research. If you are not sure, read the appropriate sections before making your selection.

- [x] Life sciences
- [ ] Behavioural & social sciences
- [ ] Ecological, evolutionary & environmental sciences

For a reference copy of the document with all sections, see nature.com/documents/nr-reporting-summary-flat.pdf

Life sciences study design

All studies must disclose on these points even when the disclosure is negative.

Sample size	Triplicate measurement is gold standard for the characterization of aptamer binding interactions and allows the determination of of sample-to-sample variance. We followed the conventional gold standard and conducted triplicate measurements for all binding experiments.
Data exclusions	Several outlier points were discarded from the MST assays. The points were removed when either the initial capillary fluorescence was more than 20% different than the average capillary fluorescence, or when the MST signal indicated aggregation.
Replication	All experiments were replicated reliably on several different days. Experiments that were conducted in triplicate, or over a range of concentrations, were replicated with a minimum of n=1 and/or with a subset of the concentrations. Although all experiments showed the same trends, we did notice differences in absolute RFU values when re-labeling a new batch of protein or when switching flow cytometer instruments. Therefore, special care was used to only use a single flow cytometer instrument, and to use a single batch of labeled protein for each set of experiments.
Randomization	Each sample was tested under the same experimental conditions. The in-vitro tests were conducted in a well-controlled environment so no randomization was required.
Blinding	No blinding was required as the identity of each sample was known prior to each measurement

Reporting for specific materials, systems and methods

We require information from authors about some types of materials, experimental systems and methods used in many studies. Here, indicate whether each material, system or method listed is relevant to your study. If you are not sure if a list item applies to your research, read the appropriate section before selecting a response.

Materials & experimental systems

n/a	Involved in the study	
☐	☒	Antibodies
☐	☒	Eukaryotic cell lines
☒	☐	Palaeontology and archaeology
☒	☐	Animals and other organisms
☒	☐	Human research participants
☒	☐	Clinical data
☒	☐	Dual use research of concern

Methods

n/a	Involved in the study	
☒	☐	ChIP-seq
☐	☒	Flow cytometry
☒	☐	MRI-based neuroimaging

Antibodies

Antibodies used	Goat anti-rat IgG H&L (FITC). Supplier: abcam Product number: ab6840 Lot #: GR3376732
Validation	No in-house validation was performed to confirm antibody binding since we were not using the antibody in that capacity (we were only assessing the ability of our aptamer to bind to the well-known antibody glycosylation sites). The antibody has been referenced by at least 24 publications and has been validated for use in flow cytometry, immunomicroscopy, ICC/IF, IHC-P, IHC-Fr, and ELISA by the supplier.

Eukaryotic cell lines

Policy information about cell lines

Cell line source(s)	Dictyostelium discoideum strain AX2 was obtained by dictybase.org (Strain ID #DBS0238585)
Authentication	None of the cell lines were authenticated
Mycoplasma contamination	Cell lines were not tested for mycoplasma contamination

Commonly misidentified lines<br>(See ICLAC register)	No commonly misidentified cell lines were used in this study

Flow Cytometry

Plots

Confirm that:

- [x] The axis labels state the marker and fluorochrome used (e.g. CD4-FITC).
- [x] The axis scales are clearly visible. Include numbers along axes only for bottom left plot of group (a 'group' is an analysis of identical markers).
- [x] All plots are contour plots with outliers or pseudocolor plots.
- [x] A numerical value for number of cells or percentage (with statistics) is provided.

Methodology

Sample preparation	No cells or biological samples are used. Beads expressing aptamer sequences were created via emulsion PCR as outlined in the methods section.
Instrument	For sorting beads a BD FACSAria III Cell Sorter was used (model #648282-01). For analysis of binding assays, a BD Accuri C6 Plus Flow Cytometer was utilized (model #660517).
Software	For sorting beads, BD FACSDiva software (version 8.0.2) was used. For analysis of binding assays, BD Accuri C6 Plus Software (version 1.0.23.1) was used.
Cell population abundance	This is not relevant because no cells were used in the flow cytometry experiments
Gating strategy	The gating strategy is shown in Fig. 1 and discussed in the sorting sections of supplemental information provided. In short, the singlet bead populations are identified by the forward and side-scatter plot, and then the top ~0.1%-0.2% of the single bead population in the channel corresponding to the labeled protein of interest is collected. No cells were used in this study, so there is no need to identify positive or negative cell staining populations.

- [x] Tick this box to confirm that a figure exemplifying the gating strategy is provided in the Supplementary Information.
